# A Smartphone Step Counter Using IMU and Magnetometer for Navigation and Health Monitoring Applications

**DOI:** 10.3390/s17112573

**Published:** 2017-11-08

**Authors:** Maan Khedr, Nasser El-Sheimy

**Affiliations:** 1 Department of Geomatics Engineering, University of Calgary, 2500 University Dr NW, Calgary, AB T2N1N4, Canada; elsheimy@ucalgary.ca; 2 Department of Computer Engineering, Arab Academy for Science and Technology, Alexandria, P.O. Box 1029, Egypt

**Keywords:** adaptive step detection, step counting, activity tracking, Pedestrian Dead Reckoning, Magnetometer fusion, MEMS IMU

## Abstract

The growing market of smart devices make them appealing for various applications. Motion tracking can be achieved using such devices, and is important for various applications such as navigation, search and rescue, health monitoring, and quality of life-style assessment. Step detection is a crucial task that affects the accuracy and quality of such applications. In this paper, a new step detection technique is proposed, which can be used for step counting and activity monitoring for health applications as well as part of a Pedestrian Dead Reckoning (PDR) system. Inertial and Magnetic sensors measurements are analyzed and fused for detecting steps under varying step modes and device pose combinations using a free-moving handheld device (smartphone). Unlike most of the state of the art research in the field, the proposed technique does not require a classifier, and adaptively tunes the filters and thresholds used without the need for presets while accomplishing the task in a real-time operation manner. Testing shows that the proposed technique successfully detects steps under varying motion speeds and device use cases with an average performance of 99.6%, and outperforms some of the state of the art techniques that rely on classifiers and commercial wristband products.

## 1. Introduction

Recent advances in Micro-Electro-Mechanical Systems (MEMS) technology has made it feasible to manufacture Inertial Measurement Units (IMU) sensors that are low cost, low on power consumption on chip and also lightweight [1,2]. Most of the smart devices pedestrians use these days are enabled with such technology. The presence of those sensors at the disposal of the user make them very appealing to be used for various applications such as activity and health monitoring [3], gaming and personal navigation [4], and emergency services [5].

An IMU is a group of sensors that can sense the motion of the user represented as accelerations and angular rate changes of orientation. MEMS IMUs are low-grade sensors that are also referred to as commercial-grade. In [6], a comparison of the different types and grades of IMU is presented. The comparison shows the relatively high errors of the commercial-grade IMUs in comparison to the higher more expensive counterparts. In [7], VecorNav—one of the leading inventors of embedded navigation solutions—presents a comparison between the performance of the different grades of IMUs, and the expected deterministic errors of each of them. In addition, Reference [8] provides a performance comparison between the different underlying technologies used in IMU sensors. The performance charts show that MEMS technology suffers from the highest error budget. On the other hand, it is not practical for a user to use those high-grade IMUs for navigation as they tend to be expensive, big, heavy, and require higher power sources, and they cannot be installed on smartphones.

Applications that attempt to track pedestrian motion and activity for health purposes usually require an accurate step detection technique. Step detection is of importance in health monitoring applications where acceleration is the most exploited measurements for step detection [9]. Step count can be used to assess the physical activity level of the user, providing feedback and motivating a more active life style [10,11]. In [12], a list of some of the recent wearable technologies that provide acceleration measurements and step counts is presented in light of showing the importance of such devices in activity monitoring, and how the medical society can benefit from them. Objective physical activity assessment require information regarding the type of motion taken and its effect; some of those factors are the speed and displacement of motion [13]. Wireless Body Sensor Network (BSN) is a group of wearable sensor nodes with computational, storage, and wireless transmission capabilities that are allocated on different body parts to monitor body motion, skin temperature, heart rate, and more [14]. Other simpler approaches based on a single node such as smartwatches and fitness bands are also available, where they rely on pedometry concept. There is a trade-off between the two approaches, where the former one is more accurate and provide a lot of information that is useful for assessing the overall health condition of the user but they are too complicated and expensive, and the later one is much simpler and cheaper but less accurate.

Another category of applications that can be step-based is navigation applications. The primary source for tracking a user’s location is the Global Navigation Satellite Systems (GNSS). GNSS systems are always undergoing modernizations that increase their coverage and accuracy, and, although it can provide reliable positioning and navigation information in most cases, it suffers from degraded service in environments where the satellite signals are blocked, attenuated, or reflected [15]. Such environments include urban canyons and indoors, where most of our day-to-day activities take place. An alternative technology to GNSS is Inertial Navigation Systems (INS). Recent advances in INS [16] make them appealing for the use as an aiding for the GNSS in the case of an outage or unreliable signal, and they can also be very useful in the case of pedestrian navigation. When GNSS services are degraded, the IMU measurements can be used for over a short period of time to aid the GNSS in providing a continuous navigation solution that works under varying conditions. This integration is referred to as coupling, and it can be in one of two forms: loosely-coupled, or tightly-coupled [6,17].

With the high error magnitude and noise of MEMS IMU comes a limitation to their reliability over time. The use of conventional Dead Reckoning (DR) methods tends to drift quickly over short time span, yielding wrong navigational information. Methods for correction have been proposed and used to enhance performance such as Zero Velocity Update (ZUPT) [18], Zero Angular Rate Update (ZARU) [17], Magnetic Angular Rate Update (MARU) [19], and Heuristic Drift Reduction (HDR) [20]. The problem with these techniques is the requirement of certain conditions to be met before they can be applied, which in most cases does not hold with handheld devices.

Pedestrian Dead Reckoning (PDR) is a special form of Dead Reckoning (DR) which exploits information about the human motion, namely the Gait Cycle (GC) [21] to limit the drifting of the solution. PDR is composed of three main algorithms, step detection, step length estimation, and alignment, where the user location is obtained through the accumulation of steps, given a step length and the direction of each step [22]. Hence, step detection is a crucial component of PDR.

The remainder of this paper is organized as follows: Section 2 presents the related work and state of the art techniques for step detection. Section 3 discusses the proposed new methodology for the step detection. Section 4 presents the experimental setup for testing the hypothesis, and the results of the algorithm. Finally, Section 5 draws conclusions from the presented results.

## 2. Background

This section presents some of the existing step detection and counting techniques. The techniques presented can be categorized into two categories: strapdown systems and handheld devices. In some research, the step detection device will be referred to as a pedometer.

### 2.1. Strapdown Systems

Multiple approaches rely on the use of an IMU strapped down to a segment of the body. The advantage of such systems is the elimination of separate platform motion from the body, where nearly all of the measurements captured by the system represent the motion of the body segment it is connected to. Some of the developed systems are: foot mounted, waist belt mounted, or wrist mounted. The most exhaustively tested approach for navigation purposes is foot mounted.

Foot mounted approach relishes the benefits of closely capturing the characteristics of the GC and the underlying kinetics of it. For example, during the stance phase, it is expected that the IMU is stationary, hence it is deduced that the measurements from the accelerometer tend to gravity value, while the gyroscope value tends to zero. Exploiting this information, the stance phase can be easily detected. Furthermore, the accurate detection of the stance phase would enable the use of correction methods for compensating for the drift in the measurements. For the case of foot mounted sensors, the detection process relies on the use of thresholds for detecting the stance phase in most cases, or the use of GC state transition modeling.

A threshold-based approach that relies on the use of gyroscope measurements for the detection of the stance phase is proposed in [23]. The stance is detected when the gyroscope measurements fall within a predefined threshold. A more reliable approach presented in [24] where multiple threshold-based constrains are defined for both accelerometer and gyroscope measurements, where three threshold checks are carried out and a step is identified when all three conditions are met. Another threshold-based approach is found in [25], it uses thresholds for acceleration measurements to identify a stance, but it also incorporates a validation step through defining a minimum time span for the stance.

Threshold-based detection can suffer from degraded performance especially in fast walking and running modes, where the stance phase period is diminished or eliminated. To overcome this limitation, some researches apply gait phase classifier, where the goal is to detect all GC phases to detect the cases when the stance phase is undetected.

The authors in [26] define a Finite State Machine (FSM) with a probability transition matrix to identify the four main phases of gait. The classification is based on the accelerations from a tri-axial accelerometer and a single axis gyroscope. Similarly, in [27], a state transition is defined. The update in this approach is the use of a tri-axial gyroscope instead of only a single axis gyroscope. In [28], an FSM that assumes that the GC is a Hidden Markov Model (HMM) is proposed. The measurements from the gyroscope and the force resistors installed on the sole of the shoe are used for the phase classification along with the probability transition matrix. This work was modified in [29] to replace the force resistors by accelerometer and redefining the probability transition matrix. A Bayesian Network (BN) was proposed in [30]. The aim of the network is to distinguish the stance phase only using a set of three threshold-based constraints for the accelerometer and gyroscope measurement, and a predefined GC phase threshold defined by kinesiology. In [31], a study of the effect of footwear effect on gait features is proposed. The study uses Artificial Neural Networks (ANN) for gait feature detection for the same subject under the use of different footwear types. It is shown that different types of footwear, namely: bare-foot, sneakers, and high heels, have different effects on the accelerations generated during walking. From this study, it is drawn that some external conditions might affect the performance of non-adaptive step detection techniques. More research based on ANN was presented in [32], where an ankle–foot orthosis was used. The orthosis was equipped with an IMU, two Force Sensitive Resistors (FSRs) mounted on the sole, and an angle sensor mounted vertical to the ankle. The purpose of this research was to detect steps and classify the action being taken, such as stair ascent/descent, or level ground. By detecting the type of motion, the actuators of the orthosis can be modified accordingly to facilitate the motion. 

A different approach was presented in [33] that proposes the use of a magnetometer on one shoe, while placing a permanent magnet on the other. The algorithm detects the steps through the processing of the magnetometer measurements, which are no longer the magnetic north but the proximity of the magnet on the other shoe.

Due to recent advances in wearable devices, namely smart watches [34] and fitness bands, they have attracted a lot of research in the health monitoring applications. Those devices enabled with IMU along with a variety of sensors such as heart rate, and temperature sensors. The use of wrist strapdown systems utilizes the same techniques as the foot-mounted but suffer from the disadvantage of the free motion of the arm, where the arm undergoes motion that is not related to walking behavior in some cases, and hence require more analysis. On the other hand, the wrist placement can be more beneficial for health applications for its direct contact with the skin, e.g. it can sample heart rate and skin temperature along with other useful information. Two examples of recent patents for step detection utilizing a wrist placement device can be found in [35,36]. Different kinds of pedometer implementations and placements have been compared in [37], where the results show that the most desirable place is the waist. It is also worth noting that the paper did not include foot mounted systems and only evaluated smartphone performance in a pocket placement state.

For the use of threshold-based detection and state transitions, a predictable pattern should exist. This hypothesis holds for tethered sensors in most cases such as the foot-mounted case, but, in the case of a free-moving handheld device, there are no predictable outcomes at a given time, and hence researchers resort to other methods as will be presented in the next subsection.

### 2.2. Handheld Devices

In the case of untethered-handheld devices, there is no detectable zero velocity region for the stance phase of the GC as the human upper body half is in continuous motion unlike the foot, and the device might exhibit non-walking related motion from the arm motion causing orientation changes and accelerations that do not represent the walking behavior. Hence, methods for step detection rely on peak extraction instead of zero acceleration periods. Regular peak detection techniques usually rely on classifiers to determine the use of the handheld device—smartphone—to adaptively adjust the step detection thresholds.

Peak detection has been exploited in many researches. In [3],a technique based on peak detection is proposed. It requires a training phase to estimate the user dependent thresholds for step detection before it can be used for navigational purposes. This is inconvenient, as the parameters estimated will work for one user but it is not guaranteed to work for others, while another limitation is that it only works in compassing mode.

A classifier is developed in [38] to identify the type of motion the device is experiencing. Once the device use case is identified, a decision of using either the accelerometer or the gyroscope measurements is made based on the class of motion identified. The steps are then detected through peak extraction technique. The classifier proposed is a supervised classifier, which means a training phase was required to obtain the thresholds for the decision making in the classifier.

Another approach that uses a classifier was presented in [39]. It classifies the motion type to two classes through the use a periodicity detection algorithm. Peaks are then extracted to represent candidate steps and validated for removal of false steps. The validation of candidate steps is through the integration of the measurements during the step duration to check if significant displacement occurs. In [40], a classifier that for three use cases is used, namely holding, swinging, and pocket placement. Based on the classification, the acceleration component to be used is selected, where it can be the *z*-component, *y*-component, or the vertical acceleration from the leveled measurements. A feedforward ANN with pattern recognition was proposed in [41]. The network has only directed connections and requires reference data for training. The approach used utilizes the ANN for the step detection and step length estimation.

In some use cases of the phone, fake signals that look like human motion can be simulated leading to false step counts. The authors in [42] propose an adaptive filtering method for eliminating false peaks. Unlike most approaches, the presented work uses the norm of the acceleration measurements from the accelerometer and not only vertical acceleration analysis for the step detection. The process starts by extracting pairs of peak/valley. Each peak and valley being detected is a candidate until verified through magnitude and temporal filtering. Upon the verification of a peak/valley pair, a step is identified. 

A different approach of classification is developed in [43] by employing a fuzzy-logic classifier. Features from a Band-Pass filtered acceleration norm are extracted and evaluated with a membership function. The output of the classifier is then processed through a set of rules for defuzzification to identify the step.

While there are other approaches that utilize different techniques for step detection, peak detection and threshold based techniques are usually employed due to their simplicity and low overhead. A summary of different approaches that rely on the analysis of the IMU sensor measurements analysis is found in [44]. The different approaches are evaluated on bases of complexity, computational overhead, and real-time applicability.

Other methods for step detection that rely on other sensors have been suggested such as using the camera for visual odometry. A camera-based step detection example can be found in [45]. The limitation of such approach is that the smartphone motion is restrained to count the steps, the smartphone should be held in a position that captures the foot motion. Similarly, in [46], visual odometry is utilized but with a different camera usage hypothesis. The hypothesis is that the camera orientation captures the pedestrian’s first-person perspective. The proposed methodology uses the Speeded Up Robust Features (SURF) algorithm for feature extraction from the captured frames. This approach requires holding the device in a certain way, limiting the usability of the device.

Step detection and counting is of great importance for many applications, and although tethered approaches show high accuracy, it would be more desirable to have a non-constrained device, of multiple purposes use for the user to use such as a smartphone. The dynamics of the smartphone along with the unpredictability of walking behavior changes of pedestrians make it a challenging task. The authors of this paper propose an algorithm for step detection and counting that is unified for all use cases of the smartphone and step modes of the pedestrian through using features that are invariant to both.

## 3. Methodology

Using a handheld free-moving device, such as smartphones for motion tracking, exhibits a different motion pattern from a strapdown system, such as placing an IMU on the shoe or waist belt area. One of the main differences is the absence of the static period, which is usually exploited in the foot mounted systems for step detection. The usual pattern that is expected in the case of a handheld device in a static pose is represented as a sequence of alternating peaks and valleys, where each peak/valley pair represents a single step.

In this section, a novel step detection algorithm is proposed that is independent from the smartphone use case and does not require a classifier to adaptively tune the parameters for the step detection. The algorithm is based on conclusions drawn from extensive analysis of the three signals used for the step detection which are the acceleration norm, angular rates vector, and magnetic vector.

**Figure 1:**
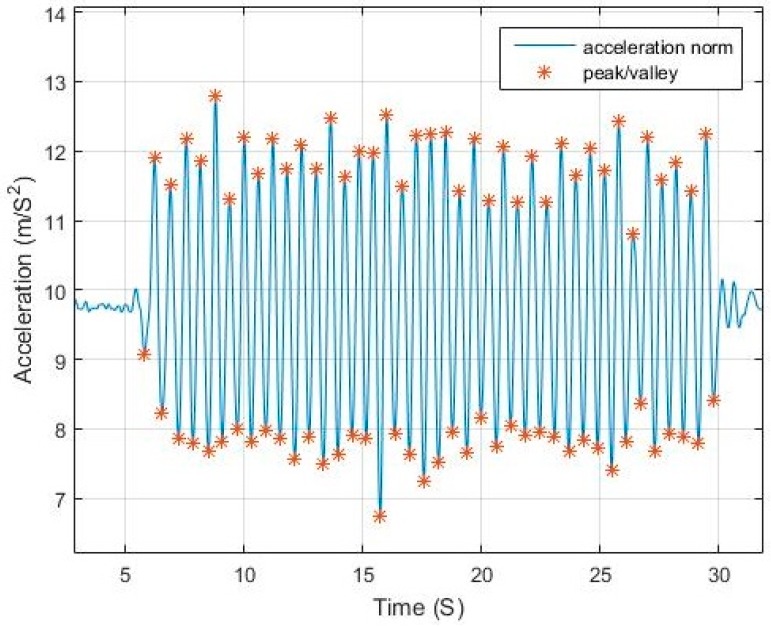
Walking sinusoidal pattern.

**Figure 2:**
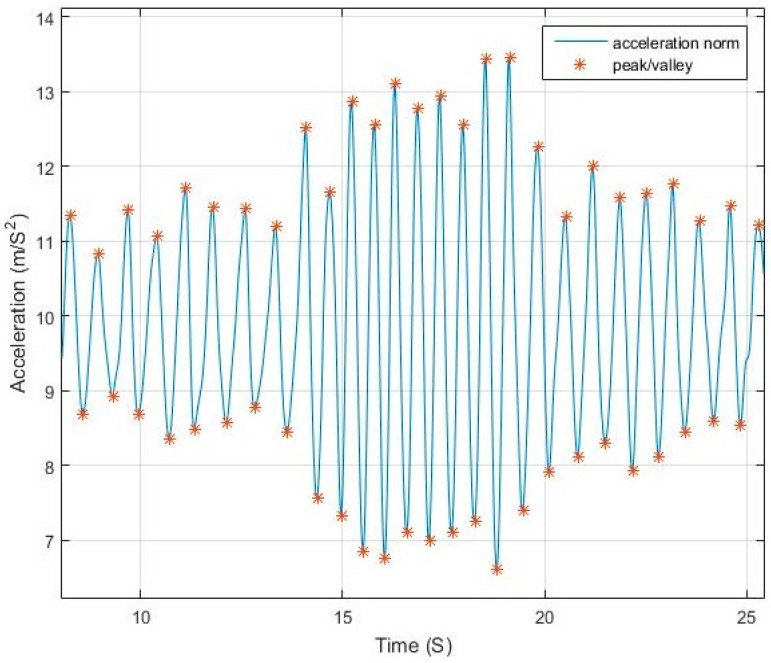
Walking with varying pace.

**Figure 3:**
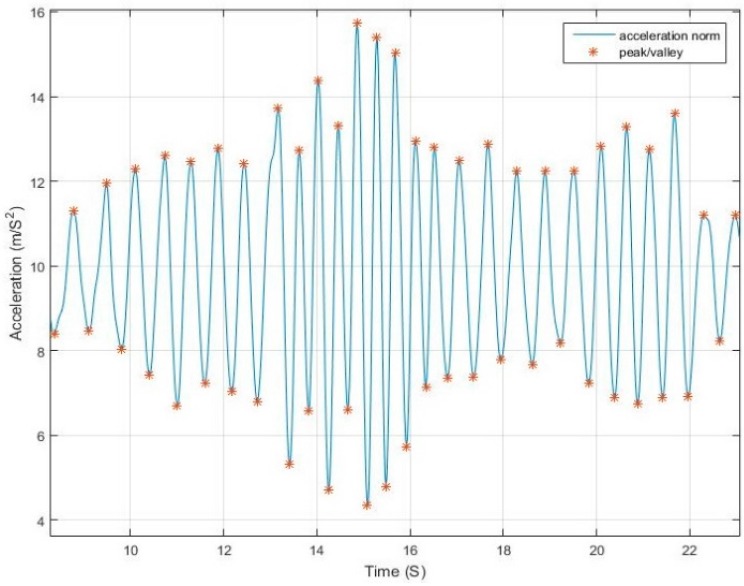
Walking and running alternation.

**Figure 4:**
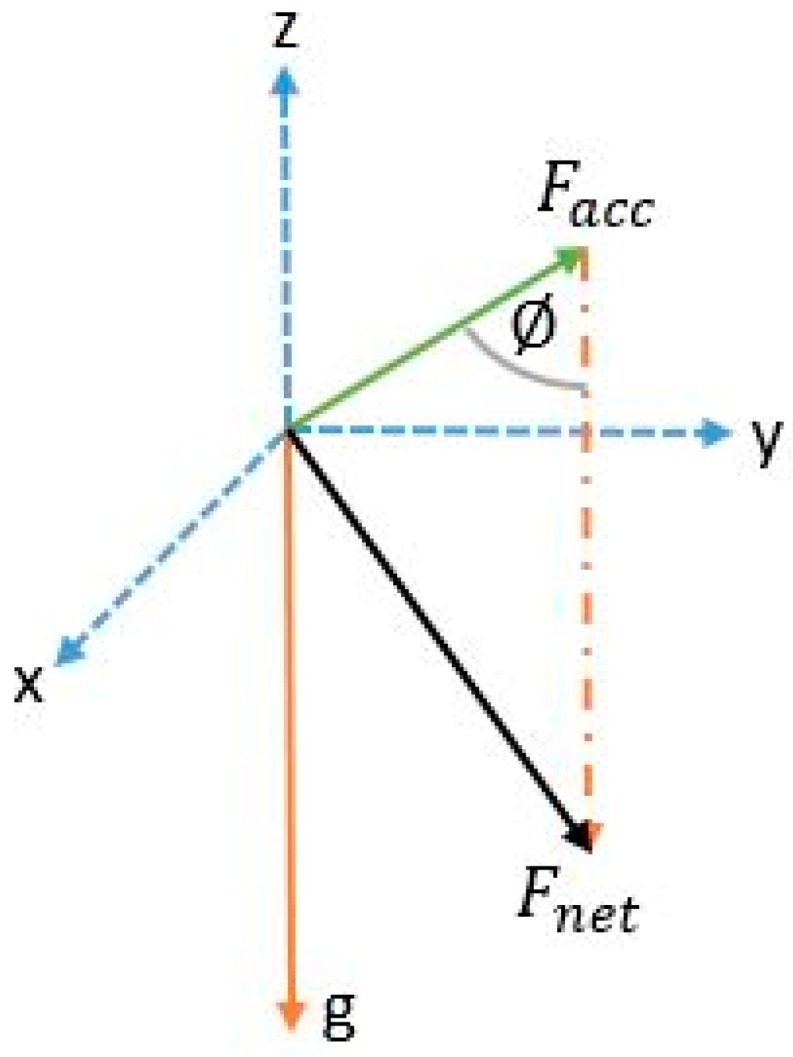
Forces applying to the accelerometer and their equivalent.

First, the acceleration norm is used without gravity compensation to avoid errors introduced from the separation process and the transformation of measurements into the Local Level Frame (LLF). From studying the norm of the accelerations in the case of a fixed device pose—compassing mode—to obtain the pattern of accelerations exerted by the walking motion, it was concluded that the acceleration norm has the following properties:

- The acceleration norm computed from Equation (1) has a sinusoidal pattern where each pair of peak/valley represents a step. Figure 1 shows an example of walking pattern with a smartphone in compassing mode with peaks and valleys detected by the proposed algorithm.
- The magnitude difference between the peak/valley pair is inversely proportional to the step duration and proportional to the motion pace. Figure 2 and Figure 3 elaborate the change in magnitude in correspondence to pace variation with time.
- The use of net force acceleration norm—uncompensated for gravity component—as shown in Figure 4, magnifies the pattern in the signal around the peaks and valleys while also smoothing it around the gravity shift component. This is due to the combined factor from both linear acceleration and gravity as presented in Equation (2).
- Although in many studies it is assumed that the measured norm is the root of sum of square of gravity and linear acceleration, from physics, the resulting force from both vectors is computed, as in Equation (2). Simply subtracting the gravity value from the resultant does not yield the linear acceleration where residuals from gravity remains due to the component derived from the angle between the vectors.

As for the case of angular rates measured by the gyroscope and the magnetic vector measured by the magnetometer, the resulting signals are useful in the phone dangling use-case. In a phone dangling state, the user holds the phone in his hand while swinging his arms in a normal motion as when walking holding nothing or something of minimal weight that does not affect his motion. In this case, the patterns generated also resemble a sinusoidal wave but each half of the signal represents a step.

(1) $$Acc=\sqrt[{2}]{{a_{x}}^{2}+{a_{y}}^{2}+{a_{z}}^{2}}$$

(2) $$Acc^{2}=Specific\;force^{2}+g^{2}-2*F_{acc}*g*\cos\left(\emptyset \right)$$

where Acc is the net acceleration magnitude; Specific force is the linear acceleration vector norm; g is the gravity norm; ∅ is the angle between gravity and linear acceleration; and $a_{x},a_{y},\;a_{z}$ are the accelerations in the body frame.

**Figure 5:**
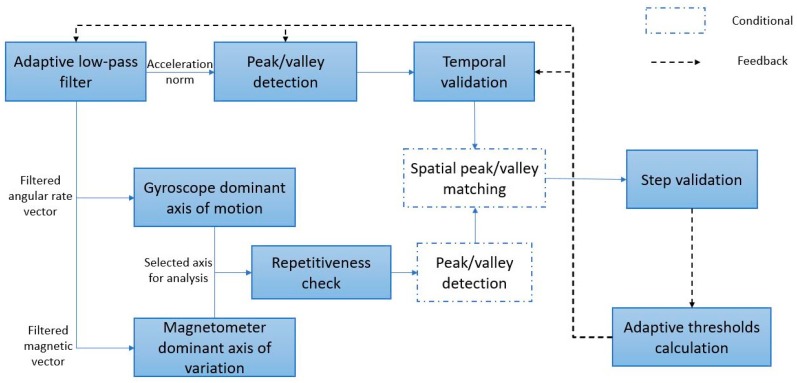
Step detection block diagram.

Based on these findings, the proposed algorithm, shown in Figure 5 as a block diagram, starts by filtering the measurements from the sensors using an adaptive low-pass filter that is discussed in Section 3.1; after that, it applies a peak/valley pair detection for the acceleration norm, with time filtering based on the peak-to-valley magnitude and peak-to-valley delay, as per Section 3.2 and Section 3.3. Verification of peaks and valleys for cases of high device motion is also applied through further investigating the dominant axis of angular rotation and magnetic change rate, as discussed in Section 3.4 and Section 3.5, where a peak/valley extraction is also applied conditionally in the case of repetitive-high-variance patterns to the gyroscope and magnetometer measurements. The peaks and valleys of the angular velocity and magnetic field should coincide within a threshold from the peaks of the acceleration. Finally, the step is verified through the integration of the acceleration measurements within the step window, as will be shown in Section 3.6. The conditional blocks are executed only in the case of the detection of a dominant repetitive signal in the gyroscope or magnetometer measurements or both.

### 3.1. Adaptive Filter

Sensor signals are poised by different errors and inaccuracies due to many factors. In the case of IMU measurements, the signals can be analyzed to compensate for deterministic errors. White noise and process noise can be hard to determine and model, hence the need for digital filtering. For a signal to be successfully filtered, the frequency of the desired signal needs to be estimated. 

The proposed system makes use of an Infinite Impulse Response (IIR) Butterworth low pass filter for its simplicity and low computational overhead. An adaptive cut-off frequency is continuously tuned and updated based on the recently detected walking speed.

Selection of the filter order is important, as it affects two main aspects of the filter, the latency and roll-off. In a Butterworth filter, the higher the order of the filter, the higher the steepness of the transition between the pass and stop bands yielding fast roll-off which makes it closer to an ideal filter, but, on the other hand, the group delay increases making real-time processing unachievable. With a low order, the latency is low but the roll of is slower and hence frequencies from the stop band still exist in the filtered signal. For the desirability of real-time processing in this application, a low filter order is needed to minimize the latency. To overcome the slow roll-off of the filter, the cut-off frequency is slightly reduced to compensate for the effect of undesired frequency residuals.

**Figure 6:**
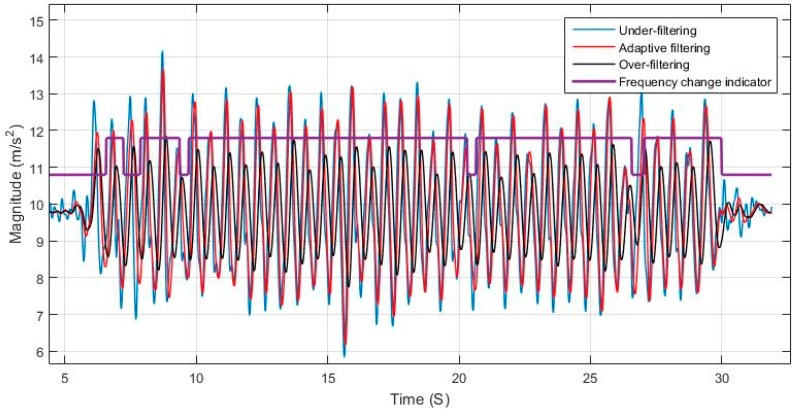
Adaptive filter comparison to constant cut-off frequency for nearly constant walking speed.

**Figure 7:**
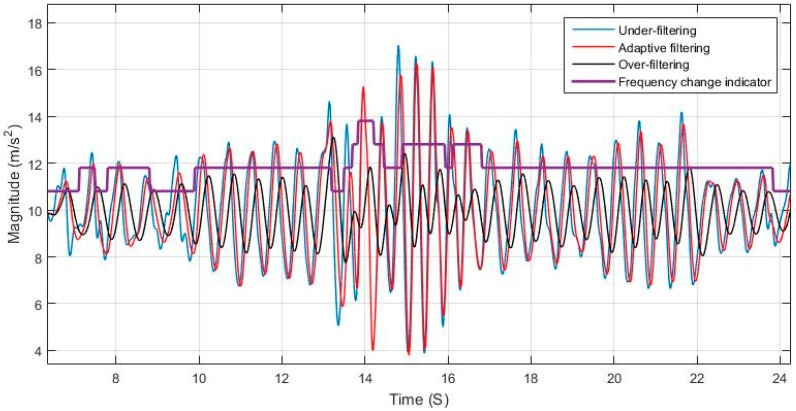
Adaptive filter comparison to constant cut-off frequency for changing walking speed.

**Figure 8:**
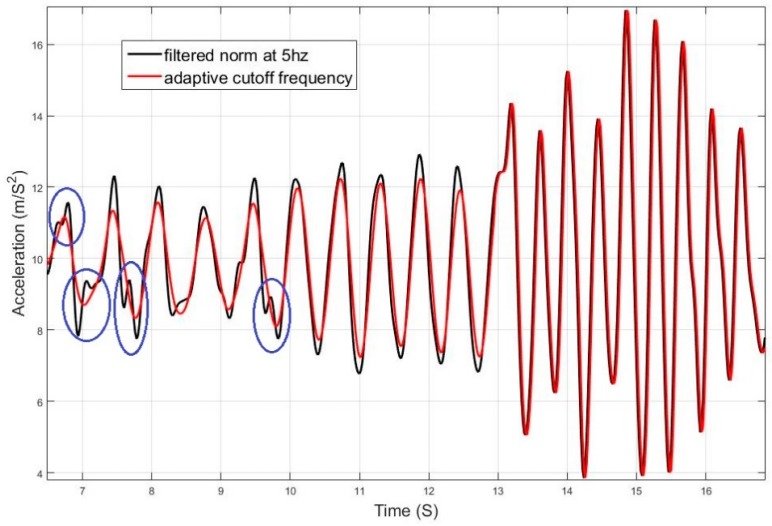
Effect of appropriately selecting the cut-off frequency of low-pass filter.

Using a static cut-off frequency for filtering a signal with varying frequency can lead to either loss of information or left over residual noise that affects the system. The effects of over and under estimating the cut-off frequency is shown in Figure 6 and Figure 7, and are compared to the case of adaptively tuning the cut-off frequency. Figure 8 elaborates on the specific effect of under-filtering in comparison to the adaptive filter as residual fluctuations from motion noise remain in the signal.

### 3.2. Temporal Filtering

Due to the noise in the signal, in some cases, two consecutive peaks or valleys can occur. The adaptive filter helps in reducing the chances of this happening, yet a fail-safe is needed to eliminate the undesired residual pikes. The temporal filtering works through adaptively tuning a time threshold, where a detected peak/valley can be replaced by another one of higher/lower magnitude. Occurrence of peak/valley outside the defined replacement zone is neglected unless it is an opposing type of peak with high magnitude difference from the recently detected peak.

**Figure 9:**
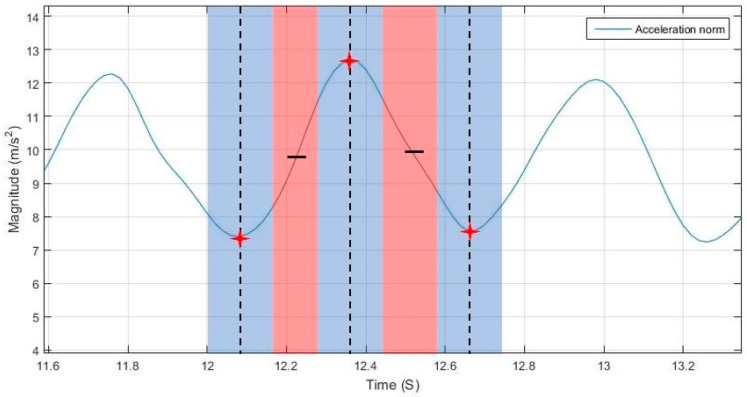
Update and rejection zones for peak/valley detection.

Originally, a peak or valley is detected if the value in the middle position of a window is greatest or lowest respectively, as shown in Equation (3), where the detected peak/valley is a candidate that is only verified if not replaced by another within a time threshold. Figure 9 shows the temporal threshold for update and rejection, where the blue region is the update region and the red is the rejection region. The update region is defined by a starting point that is based on a time threshold from the last detected peak/valley, indicating the starting point for searching for a peak and replacing it if conditions are met. The update region ending point is based on a time threshold starting from the first peak/valley detected within this update region.

The rejection region is the intermediate time in transition from peak to valley and vice versa, where no peaks are supposed to exist in regular motion. The thresholds for defining start and end of the regions are adaptively tuned based on the estimated motion speed, as shown in Equations (4)–(8).

(3) $$peak:\;a_{n-1}<a_{n}>a_{n+1}\;valley:\;a_{n-1}>a_{n}<a_{n+1}$$

(4) $$\Delta t_{1}=t_{{\left(p|v\right)}_{n-1}}-\;t_{{\left(p|v\right)}_{n-2}}$$

(5) $$th_{s}=0.5*\Delta t_{1}$$

(6) $$th_{s}<\Delta t_{2}=t_{{\left(p|v\right)}_{n}}-\;t_{{\left(p|v\right)}_{n-1}}$$

(7) $$th_{e}=0.3*\Delta t_{2}$$

(8) $$t_{{\left(p|v\right)}_{n/r}}-\;t_{{\left(p|v\right)}_{n}}<th_{e}$$

where a is the acceleration norm; $t_{\left(p|v\right)}$ is the time of peak or valley; Δt is the time interval between peak and valley; $th_{s}$ is the time threshold for start of search/update region; $th_{e}$ is the time threshold for end of update region; and $t_{{\left(p|v\right)}_{n/r}}$ is the time of replacement candidate for *n*th peak/valley.

### 3.3. Peak-to-Peak and Pseudo Zero Crossing

For each detected sequence of peak/valley or valley/peak, the difference of the magnitudes reflects the speed of motion during the step, while their average represents the pseudo zero crossing at which a step starts or ends. The difference in magnitude referred to as the peak-to-peak value is used along with the time difference between the pair to adaptively tune the cut-off frequency for the next segment of the signal.

During regular motion, when a pedestrian is speeding up or down, the change of speed does not occur instantaneously, rather increases or decreases gradually. A peak/valley magnitude difference over time represents change in walking speed, and time difference represents a half step duration. Both can be used to tune the cut-off frequency and the time thresholds for the expected upcoming peak/valley. Equation (9) represents the magnitude at which a step is declared and the start of the next coming step. Equation (11) represents the criteria for considering a change of walking pace, which leads to the application of Equation (12).

(9) $$p_{zc}=avg\left(a_{p}\;,\;a_{v}\right)$$

(10) $$\Delta a_{n}={a_{p}}_{n}-{a_{v}}_{n}\;\Delta a_{n-1}={a_{p}}_{n-1}-{a_{v}}_{n-1}$$

(11) $$\Delta a_{n}-\;\Delta a_{n-1}>\;su_{th}\;\Delta a_{n}-\;\Delta a_{n-1}<\;sd_{th}$$

(12) f˜s=⌈FΔt∗2⌉

where: $p_{zc}$ is the pseudo zero crossing; ${a_{p}}_{n}$ is the *n*th peak acceleration magnitude; ${a_{v}}_{n}$ is the *n*th valley acceleration magnitude; $\Delta a_{n}$ is the *n*th peak/valley magnitude difference; $su_{th}$ is the speed up threshold; $sd_{th}$ is the speed down threshold and is equal to −$su_{th}$; ${\tilde{f}}_{s}$ is the estimated step frequency; and F is the sampling frequency.

The peak-to-peak magnitude and the pseudo zero-crossing are also used for detecting sudden changes of motion. When a peak/valley is detected in the rejection zone, if the magnitude difference from the pseudo zero-crossing is sufficient, while the peak-to-peak is also of high magnitude, it indicates a sudden change in pace or rapid change in the device motion separately from the user. In this case, although the peak/valley is in the rejection region, it will be accepted as a candidate.

### 3.4. Gyroscope Fusion

In some use cases, the angular rates generated have a dominant repetitive pattern in one of the gyroscope axes. The signal pattern is similar to that of the acceleration norm but half its frequency. The acceleration norm represents the full motion of the platform—the pedestrian in this case—hence capturing the patterns from both right and left legs. In the case of the gyroscope, if it is placed in a shirt pocket by the torso, it will capture the sway motion of the torso, while, if it is place in a pants pocket, whether a side pocket or rear pocket, it will sense the motion of the leg it is appended to. In both cases, the generated signal is repetitive for a full stride, which is equivalent to two steps. If the phone is handheld, under regular motion conditions the arm swings forward when the opposing leg is moving forward and swings backwards when the leg nearby moves forward. Hence, the cyclic arm motion represents two steps being taken. In the previously mentioned motion cases, the generated signal has a frequency that is half that of the acceleration.

First, a dominant axis of rotational motion is determined using the variance of the signal. Then, a periodicity check is applied. If a periodic signal is found, it is used for step detection along with the acceleration, where, for each peak or valley detected in the gyroscope signal, there should exist a peak in the acceleration norm. If high angular rates exist but no periodicity, it is an indication that the device is undergoing irregular motion that does not match the walking behavior. In such a case, the gyroscope measurements are neglected and not used for detection.

**Figure 10:**
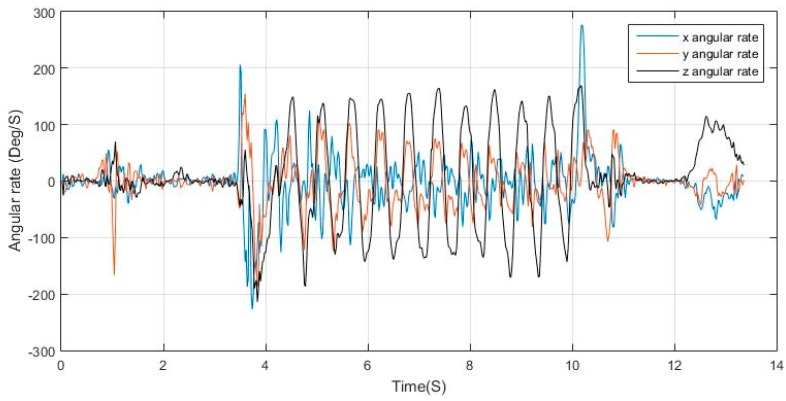
Gyroscope measurements for phone dangling case.

**Figure 11:**
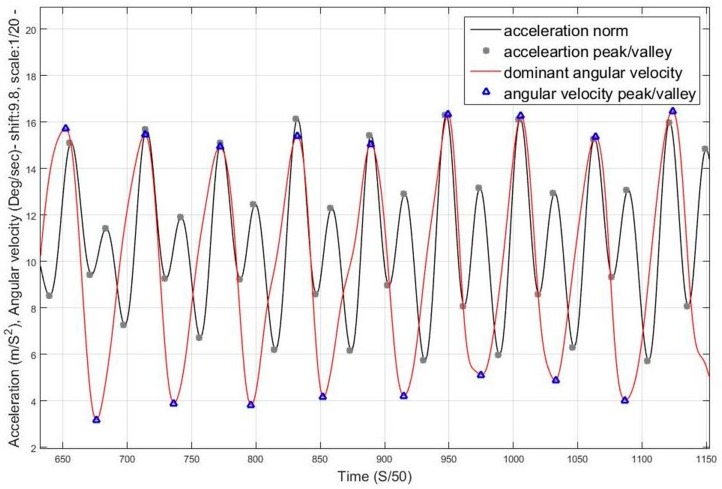
Peak matching between acceleration norm and dominant angular rate.

When the condition is stand, peak detection is applied to dominant axis of motion in the gyroscope measurements, where, for each detected peak and valley in the gyroscope, there are two corresponding peaks in the acceleration norm with a valley in between. The peak matching utilizes a time window threshold for verifying the validity of the peak. Figure 10 shows the angular rates of a regular swinging motion while walking, where it can be seen that the *z*-axis measurements are periodic with distinguishable peaks and valleys, the matching process is then elaborated in Figure 11, where, for each peak in the acceleration norm, there exists a peak/valley match in the dominant angular rate signal extracted.

### 3.5. Magnetometer Fusion

The surrounding magnetic field sensed by the magnetometer over a period is supposed to be consistent if no interference occurs. The magnetic vector has been previously used for heading estimation and for positioning using magnetic map matching. In this paper, the magnetic vector sensed by the magnetometer is used for purposes of step detection.

The hypothesis is that the magnetic field does not abruptly change within a step. Hence, any changes in the magnetic intensity measurements by the magnetometer represent a change in the orientation of the device with respect to the surrounding magnetic field.

In a dangling use case of the phone, the magnetometer axes orientation change with respect to the surrounding magnetic field resulting in a periodic signal in one or more axes of the magnetometer. This signal is similar to that generated in the gyroscope measurements and having the same properties where the signals frequency is half that of the acceleration norm of walking. For this approach to be beneficial for step detection, the magnetic change rate induced by the change of the device pose must be of higher order than the interference from surrounding sources.

**Figure 12:**
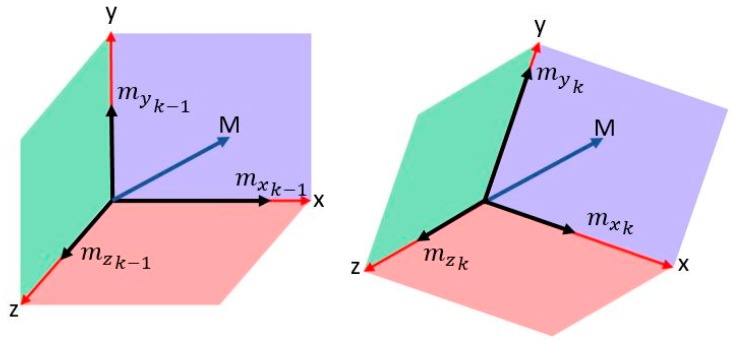
Magnetometer orientation change for a constant magnetic field and corresponding components change.

**Figure 13:**
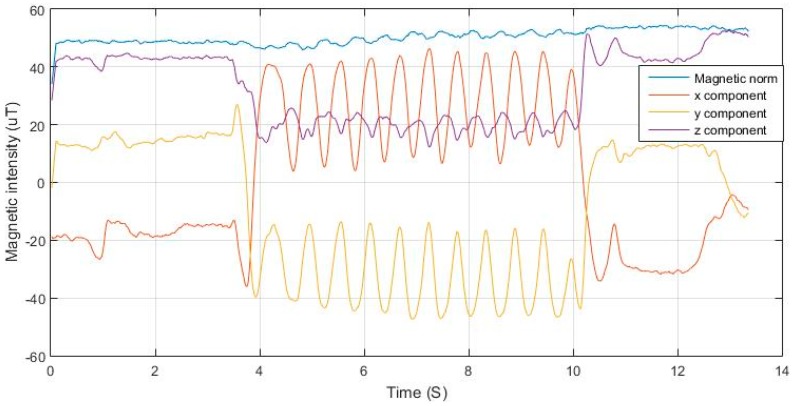
Magnetic intensity components during swinging.

**Figure 14:**
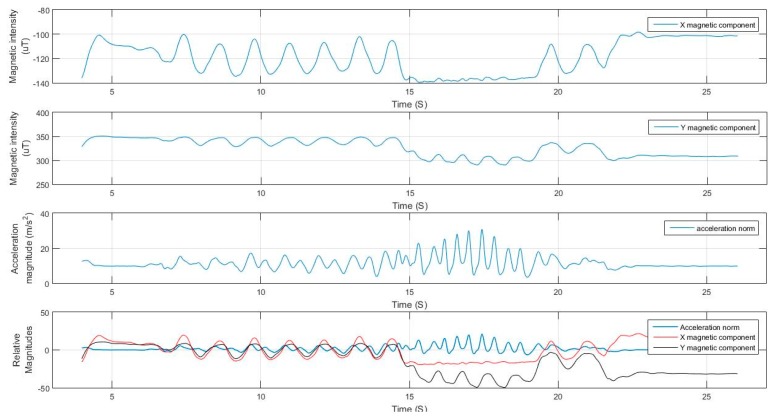
Acceleration norm and corresponding magnetic intensities for phone swinging case.

As illustrated in Figure 12, the magnetic field norm is computed based on Equation (13). As the magnetic field remains nearly constant based on Equation (14), the changes in measured components are due to changes in the orientation of the sensor frame with respect to the vector, as shown in Equation (15). Each of the axes of the sensor measure a component from the magnetic field vector depending on the non-coplanar angle between the vector and the axis based on the cosine rule. As the frame orientation changes during the motion of the user, the component will vary in a repetitive form. Figure 13 shows the variations in magnetic components in 3-D magnetometer measurements, while nearly maintaining a constant net magnitude. It is also shown in Figure 14 how the variations in the magnetic measurements coincide with the acceleration norm induced by the walking motion.

(13) $$M_{k}=\sqrt[{2}]{{m_{x}}^{2}+{m_{y}}^{2}+{m_{z}}^{2}}$$

(14) $$M_{k-1}≅M_{k}$$

(15) $${m_{x}}_{k}=M_{k}\cos\left({\theta_{x}}_{k}\right)\;{m_{y}}_{k}=M_{k}\cos\left({\theta_{y}}_{k}\right)\;{m_{z}}_{k}=M_{k}\cos\left({\theta_{z}}_{k}\right)$$

where: ${\theta_{x}}_{k},\;{\theta_{y}}_{k}\;,\;{\theta_{z}}_{k}$ are the angles from the vector to each of the body frame axes at time K $m_{x},\;m_{y},\;m_{z}$ are the magnetic intensities in the body frame; and $M_{K}$ is the magnetic norm at time K.

The peak matching for the magnetometer detected peaks follows the same rules as those of the angular rates. A magnetic peak should fall in the same window as that of the acceleration norm and angular rates. The peak matching is used to indicate magnetic perturbations, where, if an angular rate peak is found while the magnetic changes are high but has no coinciding peak, it is recognized as magnetic interference.

### 3.6. Step Validation

A step validation method is needed to verify if the detected sequence is an actual step or a mimicking behavior, where, in some use-cases, the smartphone acceleration signals with sequences of peak/valley pairs can be induced, even though the platform is not in motion. The validation in this algorithm is a simple double integration of the acceleration signals after the removal of the gravity vector to obtain the corresponding displacement for the detected pattern. The gravity can be compensated for by transforming the measurements from the sensor frame to the Local Level Frame (LLF). The algorithm proposed in [47] was used for tracking the orientation of the device to be able to obtain the measurements in the LLF where the gravity component is all summed up in the vertical axis to earth, making the separation of gravity and only obtaining the linear accelerations achievable, after which this displacement is compared to a significant motion threshold that is adaptively tuned based on the user previous steps. If the displacement is found to be greater than the threshold, the step is valid and is counted, otherwise considered as a false positive and removed. Equations (16)–(18) represent the displacement of the current step, the step threshold computation, and the condition for accepting a step. The step displacement is the double integration of the linear acceleration within the detected step period denoted by start (s) and end (e). The step threshold is computed as 0.6 of the average of the previous *k* steps.

The step window *k* is set to 3 to keep information of recent step sizes while being able to adapt to changes in walking speeds. As the window size increases, the capability of the algorithm to cope with changing walking speed would degrade at transition points with fast speed change. On the other hand, if the window is set too small, there will not be enough information to represent the motion speed when the user is walking with a nearly steady pace.

The 0.6 factor used is to compensate for sudden drop in step length when transitioning from running to walking, while neglecting displacement from accelerations integration over time from arm motion in static mode.

(16) $$d_{n}=\iint_{s}^{e}la_{norm}$$

(17) $$th_{s}=\;\frac{0.6}{k}\overset{k}{\underset{i=1}{\sum }}d_{n-i}$$

(18) $$d_{n}>\;th_{s}$$

where: $d_{n}$ = *n*th step displacement; $la_{norm}$ = linear acceleration norm; and $th_{s}$ = step displacement threshold.

### 3.7. Proposed Step Detection Algorithm

The proposed step detection and counting technique applies a sequence of algorithms to detect the steps taken by a user and verify them. The algorithm is a real-time processing of the sensor data with only a one epoch delay for detecting the peaks and valleys in the signal. Table 1 shows the notations of the variables used in the algorithm. Algorithms 1–6 show the main modules of the proposed methodology, while Algorithm 7 shows the main operation framework of the system.
**Algorithm 1.** Detect candidate (window of last three accelerations)  **If**
$\left|a_{n-1}\right|>\left|a_{n-2}\right|\;\&\&\;\left|a_{n}\right|$   **Return** (*S* = 1)  **Elseif**
$\left|a_{n-1}\right|<\left|a_{n-2}\right|\;\&\&\;\left|a_{n}\right|$   **Return** (*S* = −1)  **Else**   **Return** (*S* = 0)
**Algorithm 2.** Peak update ($th_{u}$, $S_{k-1}$, $S_{k}$)  **If**
$S_{n-1}$ == $S_{n}$   **If**
$S_{n}$ =1 && (${t_{a}}_{{peak}_{k}}$ − ${t_{a}}_{{peak}_{k-1}}$ < $th_{u}$) && ${a_{peak}}_{k}>\;{a_{peak}}_{k-1}$   **Return**(update-peak)   **Elseif**
$S_{n}$ = −1 && (${t_{a}}_{{valley}_{k}}$ − ${t_{a}}_{{valley}_{k-1}}$< $th_{u}$) && ${a_{valley}}_{k}<\;{a_{valley}}_{k-1}$   **Return**(update-valley)  **Else**   **Return**(no-update)
**Algorithm 3.** Adaptive filter frequency selector (*LPD*)  **If** previous state is static   **Return** (*Fi* = 1)  **Else**   **Return** (minimum (*Fi* = ceil(F/(LPD*2)), 6))
**Algorithm 4.** Dominant axis extraction(*ω*(*n*)*, m*(*n*))  Compute $\sigma_{\omega x}\left(n\right),\;\sigma_{\omega y}\left(n\right),\;\sigma_{\omega z}\left(n\right),\;\sigma_{mx}\left(n\right),\;\sigma_{my}\left(n\right),\;\sigma_{mz}\left(n\right)$  **For each axis**   **If** signal is periodic For all axes of gyro and mag   $d{r_{axis}}_{\omega }$ = ${\sigma_{axis}}_{\omega }/\left|2\times {f_{\omega }}_{axis}-\;f_{a}\right|$   $d{r_{axis}}_{m}$ = ${\sigma_{axis}}_{m}/\left|2\times {f_{m}}_{axis}-\;f_{a}\right|$   **Else**   dr=0  **Return** (max ($dr_{\omega }$), max ($dr_{m}$))
**Algorithm 5.** Peak matching (${t_{a}}_{peak}$, ${t_{\omega }}_{peak}$, ${t_{m}}_{peak}$)  **If**
${t_{\omega }}_{peak}$ − ${t_{m}}_{peak}$ < ${t_{th}}_{peak}$   **Return**(matching)  **Elseif**
${t_{a}}_{peak}$ − ${t_{\omega }}_{peak}$ < ${t_{th}}_{peak}$ || ${t_{a}}_{peak}$ − ${t_{m}}_{peak}$ < ${t_{th}}_{peak}$   **Return**(matching)  **Else**   **Return**(non-matching)
**Algorithm 6.** Step validation (${A_{s}}_{k}$)  $d=\iint^{\text{​}}\;{A_{s}}_{k}$ for detected step displacement  **If**
d > $th_{s}$   **Return**(valid)  **Else**   **Return**(invalid)
**Algorithm 7.** Step detection framework  Initiate adaptive filter frequencies and coefficients  Repeat for each sample:  *------- peak extraction and update section -------*  **If** in motion    Filter measurements    *S* ← Detect candidate    **If**
*S* = 1      **If**
$S_{k-1}$ = −1 && time_since_valley > $t_{a/r}$        Valid peak detected      **Elseif**
$S_{k-1}$ = *S*        $\Delta t=\;{t_{S}}_{K}-\;{t_{S}}_{K-1}$        ${a_{peak}}_{k}$ = peak update ($th_{u}$, $S_{k-1}$, $S_{k}$)        **If**
Δt ≅ step duration && significant motion detected          Account for valley miss        **Endif**      **Endif**    **Elseif**
*S* = −1      **If**
$S_{k-1}$ = 1 && time_since_peak > $t_{a/r}$        Valid valley detected      **Elseif**
$S_{k-1}$ = *S*        $\Delta t=\;{t_{S}}_{K}-\;{t_{S}}_{K-1}$        ${a_{valley}}_{k}$ = peak update ($th_{u}$, $S_{k-1}$, $S_{k}$)        **If**
Δt ≅ step duration && significant motion detected          Account for peak miss        **Endif**      **Endif**    **Endif**  *------- peak validation from gyroscope and magnetometer -------*    dominant axis extraction(*ω(n), m(n)*)     **If**
*dr* > 0        $S_{\omega }\;,\;S_{m}$ ← Detect candidate(*ω*(*n*), *m*(*n*))       **If**
$S_{\omega }$ = 1 || $S_{\omega }$ = −1        peak matching(${t_{a}}_{peak}$, ${t_{\omega }}_{peak}$, ${t_{m}}_{peak}$)        validate matching peaks      **Endif**    **Endif**  *------- step validation -------*    step validation (${A_{s}}_{k}$)    adaptive filter frequency selector (*LPD*)

## 4. Testing

To test the proposed algorithm, datasets were collected by two smartphones, namely the HTC m9 and the iPhone 6. The test scenarios explained in the following subsection were carried out on both devices by multiple users. The algorithm was implemented on the android device, which utilizes a Qualcomm Snapdragon 810 [48] with a clock speed up to 2.0 GHz. SensorLog app [49] was used on the iPhone device to log data that were processed in a sequential manner to emulate the real-time scenario. The android version operated in the background without causing degraded user experience. It is to be noted that, from the presented pseudocodes, the algorithm does not apply any extensive computations that would require high resources.

### 4.1. Experimental Setup

A group of ten users contributed to the data collection for the algorithm testing. The group is composed of five males and five females within the ages of 21–34. Each of the test subjects carried out a walking test of 100 steps for six different phone use-cases and four walking modes summing up a total of 24 tests per user. Table 2 shows the tests carried out by the test subjects.

In addition to the data presented in the previous table, six tests were carried out, where, in each test, the user walked 383–594 steps while changing the phone orientation, use-case, varying the walking speed, switching between walking and running, and scaling stairs. In those three tests, step counts provided by two wrist fitness bands were sampled to be compared with the proposed implementation. The wristbands used were the Fitbit Flex2 and the Xiaomi Mi Band 2. The bands were mounted on the right wrist while the smartphone was held in the right hand, hence all of the devices are experiencing nearly the same signals, except for the case when the phone was placed in the pocket.

### 4.2. Results

Table 3 shows the average detection success of the proposed algorithm for each combination of step mode and device pose carried out by the test subjects. The walking pace for the regular walking ranged between 1.5 and 2.3 steps, the slow walking between 0.7 and 1.3, and the running between 2.5 and 4 steps. As seen, the lowest performance recorder for step modes was in the slow walking case; this occurs because the motion noise becomes more dominant in slow step modes, like hand shaking and imbalance in motion. As the speed of motion increases, the motion noise ratio to the motion signal itself becomes less and is filtered out easily. The effect of the motion noise was highest when in texting mode, which was caused by the patters generated from the tapping on the screen being dominant and causing many fake peaks. The best reported performances where in the case of regular walking with compassing, pocket and phoning, which was due to the relative static pose of the phone, which made the motion signal dominant in comparison to the device motion.

Overall, the performance of the algorithm can be judged based on the final criterion of use-case, which is free-motion. In this test, the test subjects were asked to use the phone in a combination of different poses while walking non-stop. The most important test case is the combination of mixed step mode with free moving device. The reported accuracy is 99.6%, which means that, out of a total of 2000 steps taken by all the test subjects, only 8 steps were missed.

**Figure 15:**
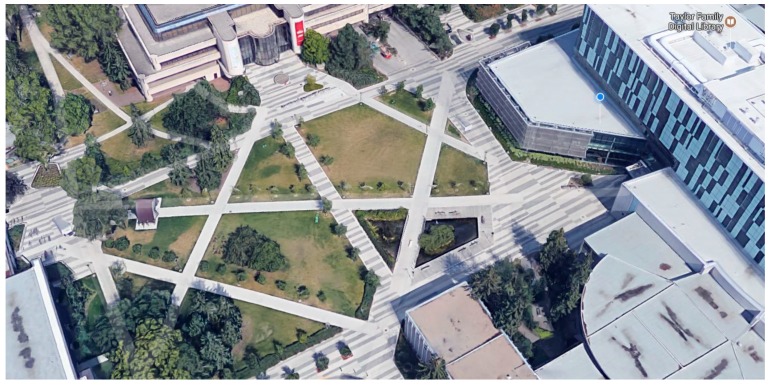
Outdoor map in front of the university library.

**Figure 16:**
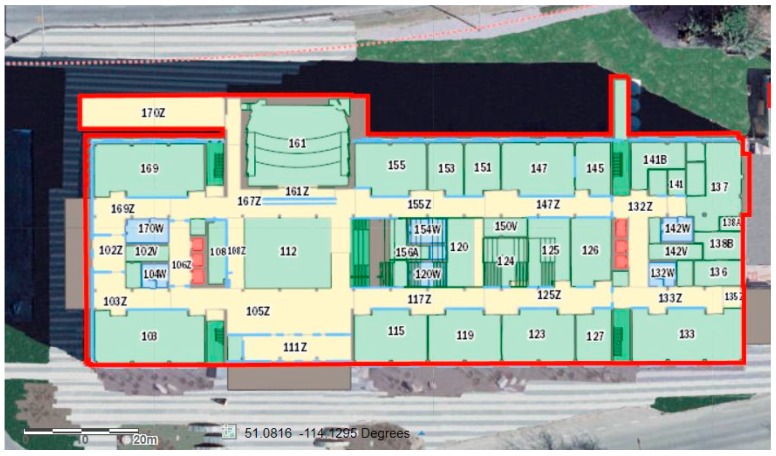
Indoor map of the Energy Environment Experimental Learning (EEEL) building where tests were carried out.

The remaining six tests were not pre-planned. For each test, the user walked around for three to four minutes switching between device pose and step modes. Table 4 shows the performance evaluation of the proposed algorithm in comparison to the FitBit Flex2 and Xiaomi MIBand2. Two of the environments where the tests were carried out are shown in Figure 15 and Figure 16. As shown, the places where the tests took place varied between indoor and outdoor environments to test the stability of the algorithm.

**Figure 17:**
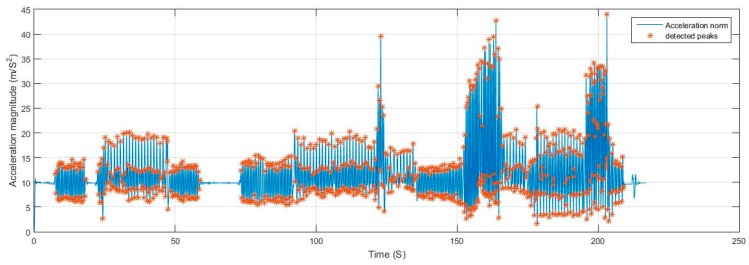
Filtered acceleration norm from Test 1 with detected peaks.

**Figure 18:**
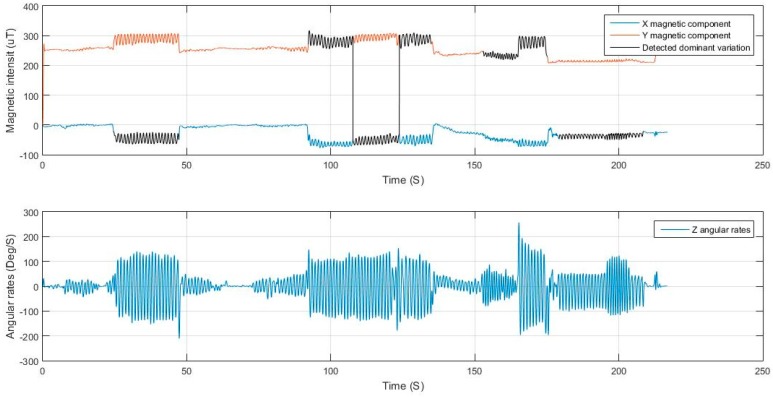
Magnetometer and gyroscope dominant axis of motion detection.

For elaboration, the signals from Test 1 are shown in Figure 17 and Figure 18. The filtering successfully adapted to the speed of motion, where at some point the user was running at a pace of 5 Hz. If the signal was filtered with a constant cut-off frequency, it would have been either under-filtered and suffer from undesired motion noise, or over-filtered where the magnitudes of the acceleration during the running period would have been drastically reduced and would have caused the system to fail in the detection.

The extraction of the dominant axis of motion for both the magnetometer and the gyroscope after filtering was also successful, where, in the case of the magnetometer, there were two dominant axes of motion and the algorithm switched between them based on the variance of the signal. It can be deduced from the figure that the user switched between dangling the phone at times and holding it nearly steady at others. The change of the orientation of the device and the step mode taken by the user did not affect the performance of the extraction nor the peak detection and hence leading to good step detection with high accuracy.

Results shown in Table 4 indicate a higher performance by the proposed algorithm in comparison to the two fitness bands used. A minimum accuracy of 99.38% and a maximum of 99.66% are reported with an average of 99.47%. In all tests, the proposed algorithm using the smartphone outperformed the accuracy of both bands.

The reported results in Table 3 and Table 4 are compensated for false positives. In some cases, in the tests, false steps were counted that should have been filtered. For each false positive detected through manual analysis of the datasets, a step was subtracted from the resulting count for that test to get the actual accuracy of detection. The case with the most reported false positives was the typing while in slow walking speed step mode with 13 false positives. The number of false positives detected was 19 steps out of 111 misdetections through the 50,836 total steps taken in the experiment. This yields a 17.12% of the miscounts being false steps, where 11.71% of the miscounts occurring in the specific combination of use-case texting and slow walking step mode.

## 5. Conclusions

A novel step detection algorithm using smartphones was proposed for detecting steps while being invariant to the device pose, use-case, and step mode of the user. An adaptive low-pass filter that continuously tunes the cut-off frequency was proposed, where the filter successfully selects the appropriate cut-off frequency reducing the motion noise in the signal while preserving crucial walking information. Fusion of information from the angular rates and magnetic intensity with the acceleration norm was proposed for peak detection verification in cases of high angular rates with periodicity. All the thresholds used for detection are adaptively computed during operation to cope with step mode variations and are independent from user specific information and behavior. The proposed algorithm shows high versatility with a maximum accuracy of 100% in some cases of fixed device pose and a minimum of 99.1% in the case of slow walking while texting due to screen tapping induced noise. The reported average accuracy is 99.6% for combined step modes with low dynamic phone pose change over time, and a 99.47% for high dynamic changes. The proposed algorithm outperformed the accuracy of two fitness bands available in the market while not requiring any extra hardware to be used, as in the case of a wrist mounted fitness band. This algorithm provides a convenient means of self-assessing activity levels while only requiring the installation of an app on a user’s smartphone, and can also impact the accuracy of a PDR by accurately detecting steps taken.

## Figures and Tables

**Table 1:** List of variable notations used in the algorithm.

Notations	
$a_{n}$	Acceleration magnitude at time *n*
${A_{s}}_{k}$	Accelerations during *K*th detected step
${a_{peak}}_{k}$	*K*th peak magnitude
${a_{valley}}_{k}$	*K*th valley magnitude
*LPD*	Last peak/valley pair difference
*F*	Sampling frequency
*Fi*	Frequency index for selecting filter coefficients, where 5 is the max index
$f_{\omega }$	Estimated angular rates frequency
$f_{m}$	Estimated magnetic intensity frequency
$f_{a}$	Estimated acceleration frequency
*S*	Peak detection state: 1 for peak, −1 for valley, and 0 for intermediate point
*ω(n)*	A window of size *n* of angular rates vector
*m(n)*	A window of size *n* of magnetic intensity vector
$\sigma_{\omega k}$	Variance for angular rates of axis *k*
$\sigma_{mk}$	Variance for magnetic intensities of axis *k*
*dr*	Dominance rank
${t_{a}}_{peak}$	Time of latest acceleration peak
${t_{a}}_{valley}$	Time of latest acceleration valley
${t_{\omega }}_{peak}$	Time of latest angular rate peak/valley
${t_{m}}_{peak}$	Time of latest magnetic intensity peak/valley
${t_{th}}_{peak}$	Time threshold for peak matching
$t_{a/r}$	Time threshold for end of rejection zone and beginning of candidate detection
$th_{u}$	Time threshold for updating acceleration peak/valley
$th_{s}$	Significant displacement threshold for step validation

**Table 2:** Collected dataset description.

Datasets				
Device Pose	Step Mode			
	Walking Regular Pace	Slow Walking	Running	Combined
**Compassing**	10 (200 steps)	10 (200 steps)	10 (200 steps)	10 (200 steps)
**Dangling**	10 (200 steps)	10 (200 steps)	10 (200 steps)	10 (200 steps)
**Texting**	10 (200 steps)	10 (200 steps)	10 (200 steps)	10 (200 steps)
**Phoning**	10 (200 steps)	10 (200 steps)	10 (200 steps)	10 (200 steps)
**Pocket**	10 (200 steps)	10 (200 steps)	10 (200 steps)	10 (200 steps)
**Free-motion**	10 (200 steps)	10 (200 steps)	10 (200 steps)	10 (200 steps)

**Table 3:** Average accuracy of the proposed algorithm.

Detection Accuracy				
Device Pose	Step Mode			
	Walking Regular Pace	Slow Walking	Running	Combined
**Compassing**	100%	99.85%	99.9%	99.9%
**Dangling**	99.85%	99.65%	99.85%	99.8%
**Texting**	99.9%	99.00%	99.9%	99.65%
**Phoning**	99.95%	99.85%	99.95%	99.9%
**Pocket**	100%	99.85%	99.95%	99.95%
**Free-motion**	99.8%	99.4%	99.75%	99.6%

**Table 4:** Performance evaluation in comparison to Flex2 and MiBand2.

Test	Performance		
	Proposed	Flex2	MiBand2
**Test 1 (383 steps)**	381–99.47%	378–98.69%	376–97.66%
**Test 2 (411 steps)**	409–99.51%	405–98.54%	407–99.02%
**Test 3 (433 steps)**	430–99.31%	426–98.38%	425–98.15%
**Test 4 (485 steps)**	482–99.38%	475–97.93%	473–97.52%
**Test 5 (530 steps)**	527–99.43%	522–98.49%	520–98.11%
**Test 6 (594 steps)**	592–99.66%	582–97.97%	579–97.47%
**Overall (2836)**	2821–99.47%	2788–98.31%	2780–98.02%
